# Supplementary material for: Platelet-Rich Plasma in Chronic Wound Management: A Systematic Review and Meta-Analysis of Randomized Clinical Trials
Source: J Clin Med. 2022 Dec 19;11(24):7532. doi: 10.3390/jcm11247532 (PMC9785167; doi:10.3390/jcm11247532)
Supplement: Supplementary file 1 [file jcm-11-07532-s001.zip › Supplement.pdf]

Supplementary Materials

F. A. Meznerics, K. D. Kovács, F. Dembrovszky, P. Fehérvári, L. V. Kemény, D. Csupor, P. Hegyi, A. Bánvölgyi. Platelet-rich plasma in chronic wound management. A systematic review and meta-analysis of randomized clinical trials.

Table S1: Summary of Findings Table

Table S2: Characteristics of the studies assessing the change of wound size

Figure S1: Forest plot for complete closure, subgrouping based on ulcer etiologies

Figure S2: Forest plot for complete closure, subgrouping based on PRP application method

Figure S3: Forest plot for complete closure, subgrouping based on follow-up time

Figure S4: Forest plot for wound area reduction, subgrouping based on ulcer etiologies

Figure S5: Forest plot for wound area reduction, subgrouping based on PRP application method

Figure S6: Forest plot for wound area reduction, subgrouping based on follow-up time

Figure S7: Risk of bias assessment of the included studies assessing the change of wound size, using the revised tool for assessing risk of bias in randomized trials (Rob 2)

Figure S8: Risk of bias assessment of the included studies assessing the change of wound size, broken down to tools, shown in percentage

Figure S9: Risk of bias assessment of the included studies assessing healing time, using the revised tool for assessing risk of bias in randomized trials (Rob 2)

Figure S10: Risk of bias assessment of the included studies assessing healing time, broken down to tools, shown in percentage

Figure S11: Risk of bias assessment of the included studies assessing infection rates, using the revised tool for assessing risk of bias in randomized trials (Rob 2)

Figure S12: Risk of bias assessment of the included studies assessing infection rates, broken down to tools, shown in percentage

Figure S13: Risk of bias assessment of the included studies assessing pain, using the revised tool for assessing risk of bias in randomized trials (Rob 2)

Figure S14: Risk of bias assessment of the included studies assessing pain, broken down to tools, shown in percentage

Figure S15: Risk of bias assessment of the included studies assessing amputation rates, using the revised tool for assessing risk of bias in randomized trials (Rob 2)

Figure S16: Risk of bias assessment of the included studies assessing amputation rates, broken down to tools, shown in percentage

Figure S17: Risk of bias assessment of the included studies assessing adverse events, using the revised tool for assessing risk of bias in randomized trials (Rob 2)

Figure S18: Risk of bias assessment of the included studies assessing adverse events, broken down to tools, shown in percentage

Figure S19: Funnel plot for complete closure

Figure S20: Funnel plot for the reduction of wound area

Supplementary References

Table S1. Summary of Findings Table.

Outcomes	№ of wounds[#] (studies)	Certainty of the evidence (GRADE)	Effect		Comments
			Relative effect (95% CI)	Absolute effect (95% CI)	
Primary outcome					
**Complete closure (№ of wounds closed) follow-up: 1 to 48 weeks**	**2198 (33 study groups of 29 RCTs)**	**⊕⊕⊕◯ Moderate**	**OR 5.32 (3.37; 8.40)**	**5 fewer per 1000** (from 8 fewer to 3 fewer)	**Higher number of completely closed wounds indicates greater improvement**
Complete closure (№ of wounds closed) etiology: venous follow-up: 1 to 48 weeks	419 (11 study groups of 9 RCTs)	⊕⊕⊕◯ Moderate	OR 8.02 (3.63; 17.71)	**8 fewer per 1000** (from 18 fewer to 4 fewer)	
Complete closure (№ of wounds closed) etiology: diabetic follow-up: 1 to 48 weeks	1442 (14 study groups of 14 RCTs)	⊕⊕⊕◯ Moderate	OR 2.26 (1.50;3.41)	**2 fewer per 1000** (from 3 fewer to 2 fewer)	
Complete closure (№ of wounds closed) application: topical follow-up: 1 to 48 weeks	1920 (28 study groups of 26 RCTs)	⊕⊕⊕◯ Moderate	OR 4.74 (2.87; 7.83)	**5 fewer per 1000** (from 8 fewer to 3 fewer)	
Complete closure (№ of wounds closed) application: injected follow-up: 1 to 48 weeks	278 (5 study groups of 5 RCTs)	⊕⊕◯◯ Low	OR 9.42 (3.32;26.76)	**9 fewer per 1000** (from 27 fewer to 3 fewer)	
Complete closure (№ of wounds closed) follow-up: <12 weeks	1157 (21 study groups of 18 RCTs)	⊕⊕⊕◯ Moderate	OR 6.03 (3.21; 11.33)	**6 fewer per 1000** (from 11 fewer to 3 fewer)	
Complete closure (№ of wounds closed) follow-up: 12 to 20 weeks	741 (8 study groups of 8 RCTs)	⊕⊕◯◯ Low	OR 3.38 (1.15;9.89)	**3 fewer per 1000** (from 10 fewer to 1 fewer)	

Outcomes	№ of wounds# (studies)	Certainty of the evidence (GRADE)	Effect		Comments
			Relative effect (95% CI)	Absolute effect (95% CI)	
Complete closure (№ of wounds closed) follow-up: >20 weeks	200 (3 study groups of 2 RCTs)	⊕○○○ Very low	OR 8.24 (1.66; 40.87)	**8 fewer per 1000** (from 41 fewer to 2 fewer)	
**Reduction of wound area (cm^2) follow-up: 1 to 48 weeks**	**1062 (18 study groups of 16 RCTs)**	**⊕⊕○○ Low**	**-**	**SMD -1.21 cm^2 lower (CI: -1.74; -0.68)**	**Lower SMD indicates greater improvement**
Reduction of wound area (cm^2) etiology: venous follow-up: 1 to 48 weeks	517 (9 study groups of 7 RCTs)	⊕⊕○○ Low	-	SMD -1.26 cm^2 lower (CI: -2.28; -0.24)	
Reduction of wound area (cm^2) etiology: diabetic follow-up: 1 to 48 weeks	201 (4 study groups of 4 RCTs)	⊕○○○ Very low	-	SMD -0.68 cm^2 lower (CI: -1.31; -0.66)	
Reduction of wound area (cm^2) application: topical follow-up: 1 to 48 weeks	694 (13 study groups of 13 RCTs)	⊕⊕○○ Low	-	SMD -0.94 cm^2 lower (CI: -1.43; -0.46)	
Reduction of wound area (cm^2) application: injected follow-up: 1 to 48 weeks	368 (5 study groups of 5 RCTs)	⊕○○○ Very low	-	SMD -1.03 cm^2 lower (CI: -1.79;-0.26)	
Reduction of wound area (cm^2) follow-up: <12 weeks	629 (4 study groups of 4 RCTs)	⊕⊕○○ Low	-	SMD -1.00 cm^2 lower (CI: -1.64; -0.35)	
Reduction of wound area (cm^2) follow-up: 12 to 20 weeks	80 (2 study groups of 1 RCT)	⊕○○○ Very low	-	SMD -1.38 cm^2 lower (CI: -2.96; 0.19)	
Reduction of wound area (cm^2) follow-up: >20 weeks	353 (5 study groups of 4 RCTs)	⊕○○○ Very low	-	SMD -0.63 cm^2 lower (CI: -1.64; 0.37)	

Outcomes	№ of wounds# (studies)	Certainty of the evidence (GRADE)	Effect		Comments
			Relative effect (95% CI)	Absolute effect (95% CI)	
Healing rate (change of wound size/time interval) follow-up: 1 to 48 weeks	453 (7 study groups of 7 RCTs)	⨁◯◯◯ Very low	not pooled	not pooled	Higher rate indicates greater improvement (see eTable 2)
Secondary outcomes					
Healing time	1198 (16 study groups of 14 RCTs)	⨁◯◯◯ Very low	not pooled	not pooled	see Table 2
Infection	1039 (16 study groups of 14 RCTs)	⨁◯◯◯ Very low	not pooled	not pooled	see Table 2
Pain	953 (11 study groups of 11 RCTs)	⨁◯◯◯ Very low	not pooled	not pooled	see Table 2
Amputation	767 (7 study groups of 7 RCTs)	⨁◯◯◯ Very low	not pooled	not pooled	see Table 2
Adverse event	1100 (15 study groups of 15 RCTs)	⨁◯◯◯ Very low	not pooled	not pooled	see Table 2

CI: confidence interval, RCT: randomized clinical trial, SMD: standardized mean difference, OR: odds ratio

Table S2. Characteristics of the studies assessing the change of wound size.

First author, year of publication	PRP application	Sample size (intervention)	Sample size (control)	Mean (SD) baseline wound area (cm^2)		Time of evaluation	Reduction of wound area				Healing rate		Comlete closure	
							Mean (SD) post-treatment wound area (cm^2)		Mean reduction of wound area (%)		Mean (SD) healing rate		Wounds completly closed (% of total)	
				Intervention group	Control group		Intervention	Control	Intervention	Control	Intervention	Control	Intervention	Control
Abd El-Mabood, 2018 [1]	topical	40	40	N/A	N/A	week 2	N/A	N/A	N/A	N/A	0.8 (0.2) cm^2/week	0.4 (0.2) cm^2/week	33.0%	10.0%
						week 4					0.9 (0.1) cm^2/week	0.5 (0.1) cm^2/week	47.5%	22.5%
						week 6					0.6 (0.9) cm^2/week	0.3 (0.2) cm^2/week	7.5%	17.5%
						week 8					0.5 (0.1) cm^2/week	0.3 (0.1) cm^2/week	5.0%	15.0%
						week 10					0.2 (0.1) cm^2/week	0.4 (0.1) cm^2/week	2.5%	10.0%
						week 12					0.19 (0.11) cm^2/week	0.4 (0.1) cm^2/week	2.5%	7.5%
						total					7.3 (0.9) cm^2/week	5.8 (0.5) cm^2/week	97.5%	82.5%
Ahmed, 2017 [2]	topical	28	28	N/A	N/A	week 2	N/A	N/A	N/A	N/A	0.7 (0.15) cm^2/week	0.5 (0.1) cm^2/week	28.5%	7.1%
						week 4					0.9 (0.1) cm^2/week	0.4 (0.2) cm^2/week	39.2%	17.8%
						week 8					0.6 (0.2) cm^2/week	0.5 (0.1) cm^2/week	14.2%	28.5%
						week 12					0.2 (0.1) cm^2/week	0.4 (0.1) cm^2/week	3.5%	14.2%
						total					N/A	N/A	85.7%	67.8%
Amato, 2020 [3]	topical	53	47	24.0 (16.0)	22.0 (9.0)	week 1	N/A	N/A	12.0%$^{+}$	10.0%$^{+}$	N/A	N/A	N/A	N/A
						week 2			23.0%$^{+}$	12.0%$^{+}$			N/A	N/A
						week 3			28.0%$^{+}$	15.0%$^{+}$			N/A	N/A
						week 4			35.0%$^{+}$	19.0%$^{+}$			N/A	N/A
						week 5			46.0%$^{+}$	22.0%$^{+}$			20.7%	4.2%
						week 6			52.0%$^{+}$	28.0%$^{+}$			26.4%	2.1%
						week 7			58.0%$^{+}$	36.0%$^{+}$			15.0%	6.3%
						week 8			64.0%$^{+}$	41.0%$^{+}$			15.0%	6.3%
						week 9			72.0%$^{+}$	49.0%$^{+}$			13.2%	10.5%
						week 10			85.0%$^{+}$	57.0%$^{+}$			3.7%	14.8%
						week 11			94.0%$^{+}$	65.0%$^{+}$			1.8%	13.9%
						week 12			100.0%$^{+}$	71.0%$^{+}$			0.0%	18.6%
						total			N/A	N/A			100.0%	68.0%
Anitua, 2008 [4]	topical	7	7	N/A	N/A	week 8	N/A	N/A	72.9%	21.5%	N/A	N/A	N/A	N/A
Burgos-Alonso, 2018 [5]	topical	7	5	N/A	N/A	week 5	N/A	N/A	39.6%	22.9%	N/A	N/A	N/A	N/A
						week 9			82.8%	40.8%			60.0%	20.0%
Cardenosa, 2017 [6]	topical	55	47	13.7 (30.0)	16.7 (23.9)	week 24	10.0 (30.0)	12.1 (19.2)	67.7%	11.2%	N/A	N/A	N/A	N/A
Chandanwale, 2020 [7]	Injected	40	40	23.0 (19.1)	22.6 (17.4)	day 8	N/A	N/A	1.4%	0.2%	N/A	N/A	N/A	N/A
						day 15	21.7	22.4	7.6%	2.4%	N/A	N/A		
						day 20	N/A	N/A	18.6%	8.2%	N/A	N/A		
						day 30	17	20	35.2%	16.2%	N/A	N/A		
						day 60	11.6 (14.0)	17.6 (15.6)	66.2%	29.9%	17.3 mm^2/day	8.9 mm^2/day		
de Oliveira, 2017 [8]	topical	9	12	7.4 (5.5-10.3)$^{+}$	16.1 (6.2-47.9)$^{+}$	week 13	2.7 (1.5-3.7)$^{+}$	4.9 (2.3-21.3)$^{+}$	64.0%	70.0%	N/A	N/A	N/A	N/A
Driver, 2006 [9]	topical	40	32	N/A	N/A	week 12	N/A	N/A	N/A	N/A	0.05 cm^2/day	0.05 cm^2/day	32.5%	28.1%
Elbarbary, 2020 (group I) [10]	topical	30	30	16.5 (8.2)	17.8 (5.4)	week 12	5.8 (2.1)	8.5 (3.3)	65.0%	52.0%	N/A	N/A	33.3%	13.3%
						week 48	1.2 (0.5)	3.8 (1.5)	92.7%	78.6%			66.7%	46.7%
Elbarbary, 2020 (group II) [10]	Injected	30	30	15.7 (7.4)	17.8 (5.4)	week 12	2.5 (1.3)	8.5 (3.3)	84.0%	52.0%	N/A	N/A	46.7%	13.3%
						week 48	1.1 (0.4)	3.8 (1.5)	93.0%	78.6%			80.0%	46.7%
Elgarhy, 2020 (group I) [11]	topical	20	20	33.7 (53.3)	15.0 (8.3)	week 6	8.5 (20.5)	10.1 (6.3)	N/A	N/A	N/A	N/A	60.0%	0.0%
						week 12	3 (7.9)	8.4 (6.6)					N/A	N/A
Elgarhy, 2020 (group II) [11]	Injected	20	20	21.8 (20.2)	15.0 (8.3)	week 6	3.2 (5.4)	10.1 (6.3)	N/A	N/A	N/A	N/A	50.0%	0.0%
						week 12	1.1 (2.3)	8.4 (6.6)					N/A	N/A
Elsaid, 2020 [12]	topical	12	12	N/A	N/A	week 20	N/A	N/A	longitudinal: 43.2%	longitudinal: 4.1%	N/A	N/A	25.0%	0.0%
									horizontal: 42.3%	horizontal: 8.2%				
Game, 2018 [13]	topical	134	134	2.29 (2.1)	2.53 (2.3)	week 20	N/A	N/A	N/A	N/A	N/A	N/A	34.0%	22.0%
Glukhov, 2017 [14]	topical	18	19	17.2 (2.8)	10.3 (5.8)	week 8	N/A	N/A	N/A	N/A	N/A	N/A	22.0%	16.0%
Goda, 2018 1 [15]	topical	25	25	7.3 (1.6)	7.08 (1.3)	week 1	N/A	N/A	N/A	N/A	0.7 cm^2/week	0.5 cm^2/week	0.0%	0.0%
						week 4							0.0%	0.0%
						week 6							0.0%	0.0%
						week 8							12.0%	0.0%
						week 10							44.0%	4.0%
						week 12							84.0%	52.0%
Goda, 2018 2 (group I) [16]	topical	10	11	<10cm^2	<10cm^2	week 2	N/A	N/A	53.4%	23.5%	N/A	N/A	N/A	N/A
						week 3			80.2%	47.2%	N/A	N/A	30.0%	0.0%
						week 4			100.0%	62.2%	N/A	N/A	100.0%	9.0%
						week 6			100.0%	100.0%	N/A	N/A	N/A	N/A
Goda, 2018 2 (group II) [16]	topical	8	7	>10cm^2	>10cm^2	week 2	N/A	N/A	31.1%	14.4%	N/A	N/A	N/A	N/A
						week 3			49.4%	29.1%			0.0%	0.0%
						week 4			64.3%	43.3%			0.0%	0.0%
						week 6			96.3%	71.6%			50.0%	14.3%
						week 7			100.0%	86.8%			100.0%	42.6%
						week 8			100.0%	97.3%			N/A	N/A
Gude, 2019 [17]	topical	66	66	4.1	5.6	week 12	N/A	N/A	N/A	N/A	N/A	N/A	48.2%	30.2%
Helmy, 2021 [18]	Injected	40	40	16.7 (11.1)	20.4 (18.5)	week 12	0.9 (2.2)	3.9 (6.9)	94.8%	80.8%	N/A	N/A	85.0%	42.5%
						week 24	0.8 (2.0)	2.2 (3.8)	95.3%	89.4%			85.0%	62.5%
						week 48	0.8 (2.1)	3.5 (6.5)	95.4%	82.6%			85.0%	47.5%
Hongying, 2020 [19]	injected	20	20	9.55 (0.61)	9.05 (1.19)	2	8.65 (0.93)	9.00 (1.21)	N/A	N/A	N/A	N/A	N/A	N/A
						4	6.85 (1.42)	8.30 (1.75)					N/A	N/A
						6	3.85 (1.89)	7.45 (2.04)					N/A	N/A
						8	1.20 (1.51)	6.65 (2.64)					100.0%	35.0%
Kakagia, 2007 [20]	topical	17	17	28.4 (13.6)	25.8 (15.2)	week 8	N/A	N/A	N/A	N/A	N/A	N/A	11.8%	11.8%
Karimi, 2016 [21]	topical	25	25	7.0 (9.8)	13.2 (9.4)	week 3	2.7 (5.9)	11.9 (13.7)	N/A	N/A	N/A	N/A	36.0%	40.0%
Kulkarni, 2019 [22]	topical	25	25	13.29 (1.3)	13.4 (1.3)	week 3	7.5 (1.0)	11.5 (1.1)	43.4%	14.0%	N/A	N/A	N/A	N/A
Li, 2015 [23]	topical	59	59	4.1 (1.4-11.4)$^{+}$	2.9 (1.0-10.5)+	week 12	N/A	N/A	N/A	N/A	N/A	N/A	84.8%	69.0%
Milek, 2019 [24]	topical	50	50	3.7 (1.0)	3.7 (1.1)	week 4	0.8 (0.4)	1.7 (0.5)	N/A	N/A	N/A	N/A	56.0%	N/A
Mohammad, 2017 [25]	topical	25	25	12.8 (14.9)	14.2 (8.52)	week 1	7.0 (9.8)	13.2 (9.53)	N/A	N/A	N/A	N/A	N/A	N/A
						week 2	5.1 (8.5)	12.3 (11.0)						
						week 3	2.7 (5.9)	11.9 (13.7)						
Moneib, 2018 [26]	topical	20	20	8.0 (16.9)	2.9	week 6	3.1 (5.9)	2.8 (1.5)	67.6%	13.7%	N/A	N/A	35.0%	0.0%
Obolenskiy, 2014 [27]	topical	44	44	90.2 (14.1)	79.6 (12.3)	week 13	N/A	N/A	N/A	N/A	N/A	N/A	85.0%	12.0%
Obolenskiy, 2017 [28]	topical	50	50	19.2 (3.0)	21.7 (2.5)	N/A	N/A	N/A	N/A	N/A	0.2 cm^2/week	N/A	92.0%	60.0%
Pu, 2019 [29]	topical	21	30	6.3 (5.3)	5.2 (5.9)	week 24	3.3 (4.2)	2.6 (8.7)	9.5%	23.3%	N/A	N/A	N/A	N/A
Qin, 2019 [30]	topical	30	30	N/A	N/A	week 12	N/A	N/A	52.1%	21.3%	N/A	N/A	N/A	N/A
	Injected	30	30			week 12			93.2%	21.3%				
Rainys, 2019 [31]	topical	35	34	12.9 (16.6)	10.4 (11.3)	week 8	6.2 (6.9)	6.9 (8.8)	52.4%	33.4%	N/A	N/A	25.7%	17.6%

**Ramos-Torrecillas, 2015 (group I) [32]**	topical PRP (1 dose)	34	25	N/A	N/A	week 5	N/A	N/A	48.3%	10.3%	N/A	N/A	9.0%	0.0%
**Ramos-Torrecillas, 2015 (group II) [32]**	topical PRP (2 doses)	25	25	N/A	N/A	week 5	N/A	N/A	54.8%	10.3%	N/A	N/A	32.0%	0.0%
**Ramos-Torrecillas, 2015 (group III) [32]**	topica PRPl+HA	40	25	N/A	N/A	week 5	N/A	N/A	80.4%	10.3%	N/A	N/A	37.5%	0.0%
**Saad Setta, 2011 [33]**	topical	12	12	10.3 (5-21)	8.5 (4-20)	week 20	N/A	N/A	N/A	N/A	N/A	N/A	100.0%	75.0%
**Saha, 2020 [34]**	Injected	56	52	5.9 (4.6)	5.6 (3.7)	week 2	3.8 (3.5)	4.9 (3.3)	39.0%	12.5%	N/A	N/A	0.0%	0.0%
						week 4	2.5 (2.6)	3.7 (2.6)	59.6%	33.6%				
						week 6	1.7 (1.8)	2.6 (1.9)	73.3%	54.1%				
						week 8	1.0 (1.1)	1.2 (1.0)	82.6%	79.3%				
						week 10	0.6 (0.7)	1.2 (1.0)	91.1%	79.8%				
**Semenič, 2018 [35]**	topical	30	30	N/A	N/A	week 24	N/A	N/A	35.0%	90.0%	N/A	N/A	N/A	N/A
**Senet, 2003 [36]**	topical	8	7	13.8 (7.9)	10.9 (8.4)	week 12	N/A	N/A	26.2%	15.2%	0.0033 (0.0061) cm/day	0.0021 (0.0058) cm/day	12.5%	14.3%
**Singh, 2018 [37]**	Injected	29	29	N/A	N/A	week 4	N/A	N/A	N/A	N/A	N/A	N/A	100.0%	92.0%
**Singh, 2021 [38]**	Injected	26	26	37.0	36.4	week 6	N/A	N/A	N/A	N/A	N/A	N/A	0.0%	0.0%
**Sokolov, 2017 [39]**	topical	83	83	N/A	N/A	week 8	N/A	N/A	N/A	N/A	N/A	N/A	34.9%	0.0%
						week 12							28.9%	8.5%
						total							63.9%	8.5%
**Somani, 2017 [40]**	topical	9	6	8.1 (5.4)	4.9 (4.7)	week 4	1.7 (3.2)	3.3 (4.4)	85.5%	42.7%	N/A	N/A	0.0%	0.0%
**Tsachiridi, 2019 [41]**	topical	15	12	0.2 (0.1)	0.5 (0.5)	week 5	0.1 (0.01)	1.5 (1.3)	N/A	N/A	N/A	N/A	0.0%	0.0%
**Tsai, 2019 [42]**	topical+injected	14	14	N/A	N/A	week 4	N/A	N/A	>75%	50-75%	N/A	N/A	N/A	N/A
**Ucar, 2020 [43]**	topical	30	30	4.7 (1.8)	4.8 (1.3)	week 8	2.8 (2.4)	5.0 (1.3)	N/A	N/A	N/A	N/A	N/A	N/A
**Yang, 2017 [44]**	topical	38	38	N/A	N/A	week 4	N/A	N/A	84.6%	77.1%	N/A	N/A	87.5%	75.0%
**Yuvasri, 2020 [45]**	topical	10	10	4.9 (3.1)	8.3 (6.7)	week 4	0.6 (1.0)	5.0 (1.3)	86.0%	72.0%	N/A	N/A	40.0%	0.0%

SD-standard deviation, PRP-platelet-rich plasma

$^{+}$ median (interquartile range)

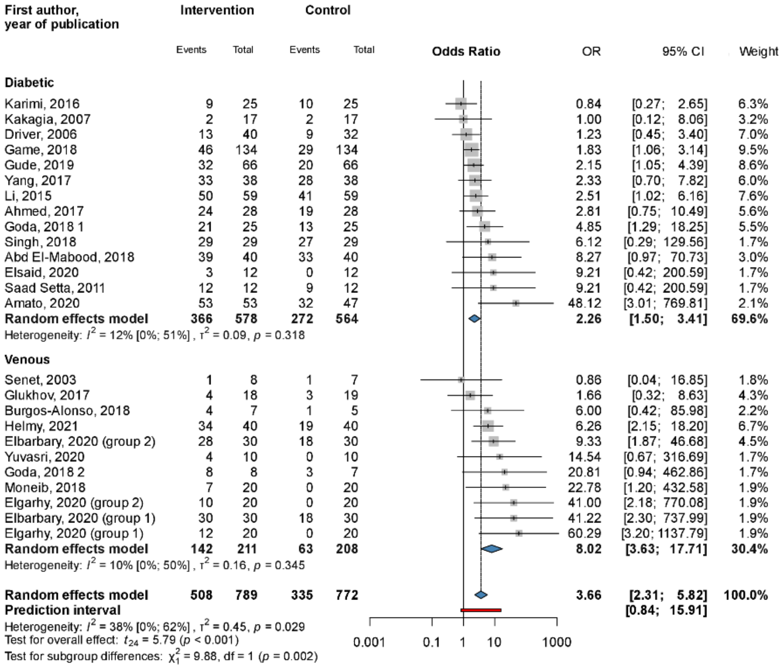

OR-odds ratio; CI-confidence interval

Figure S1. Forest plot for complete closure, subgrouping based on ulcer etiologies.

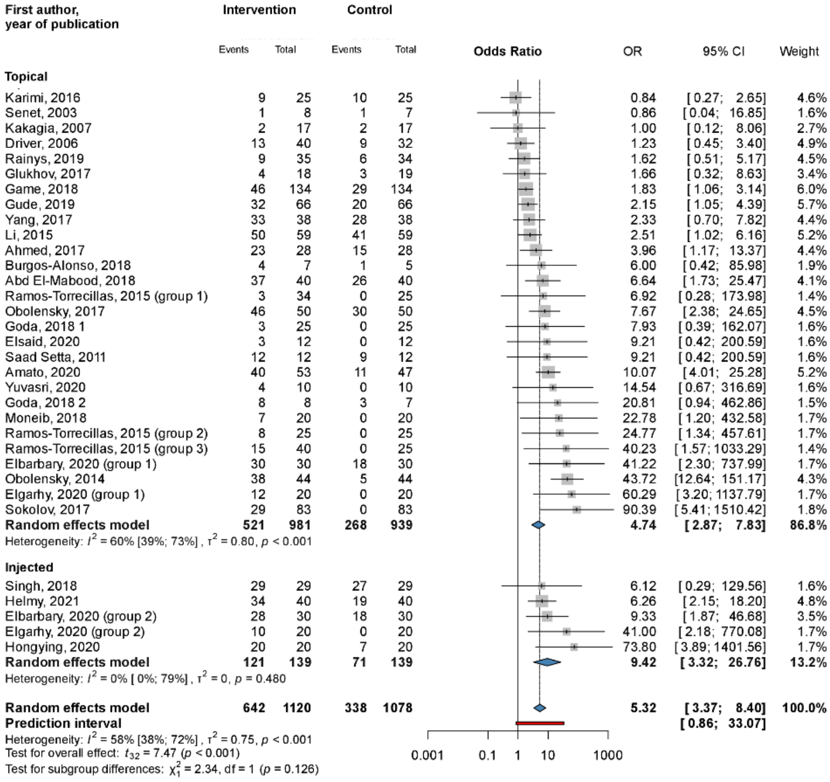

OR-odds ratio; CI-confidence interval

Figure S2. Forest plot for complete closure, subgrouping based on PRP application method.

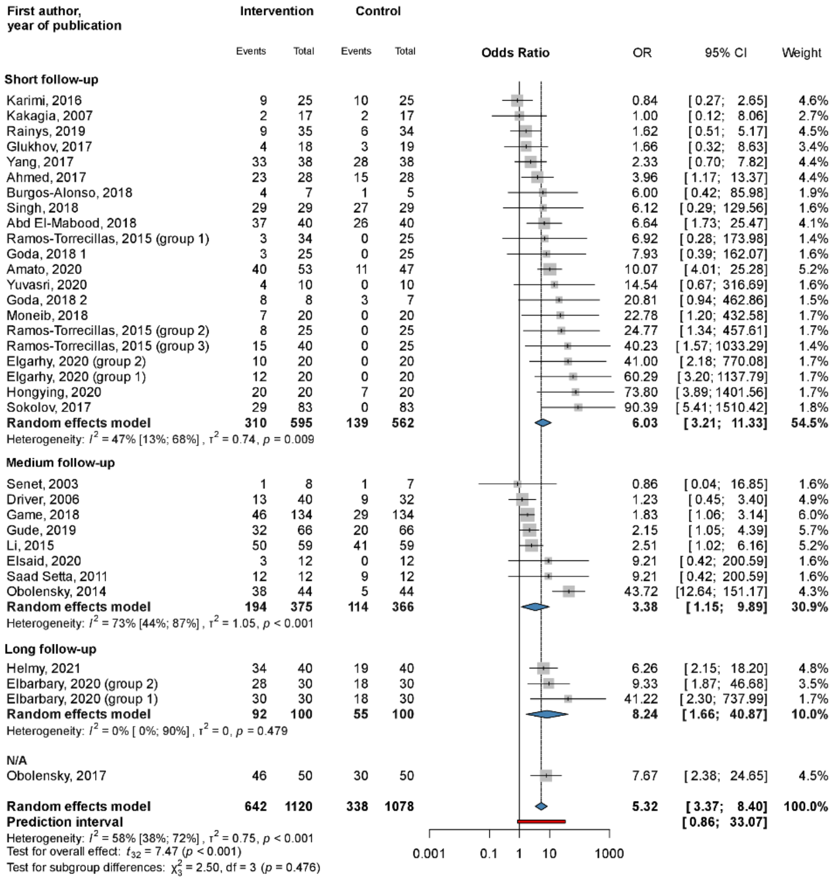

OR-odds ratio; CI-confidence interval

Figure S3. Forest plot for complete closure, subgrouping based on follow-up time.

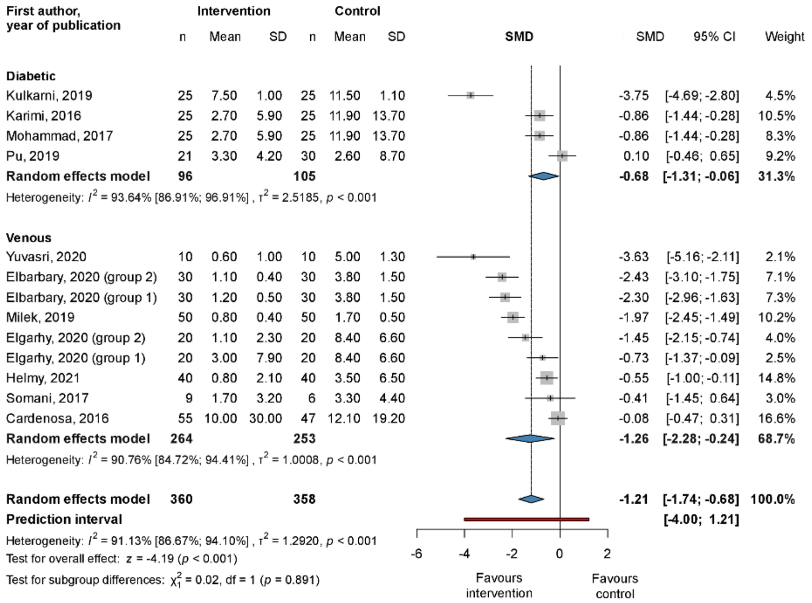

SD-standard deviation; SMD-standardized mean difference; CI-confidence interval

Figure S4. Forest plot for wound area reduction, subgrouping based on ulcer etiologies.

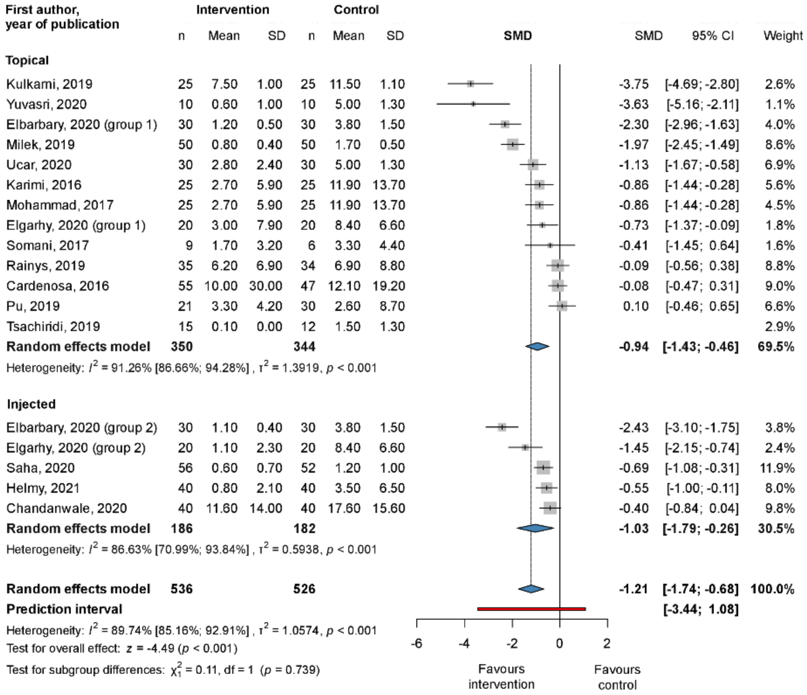

SD-standard deviation; SMD-standardized mean difference; CI-confidence interval

Figure S5. Forest plot for wound area reduction, subgrouping based on PRP application method.

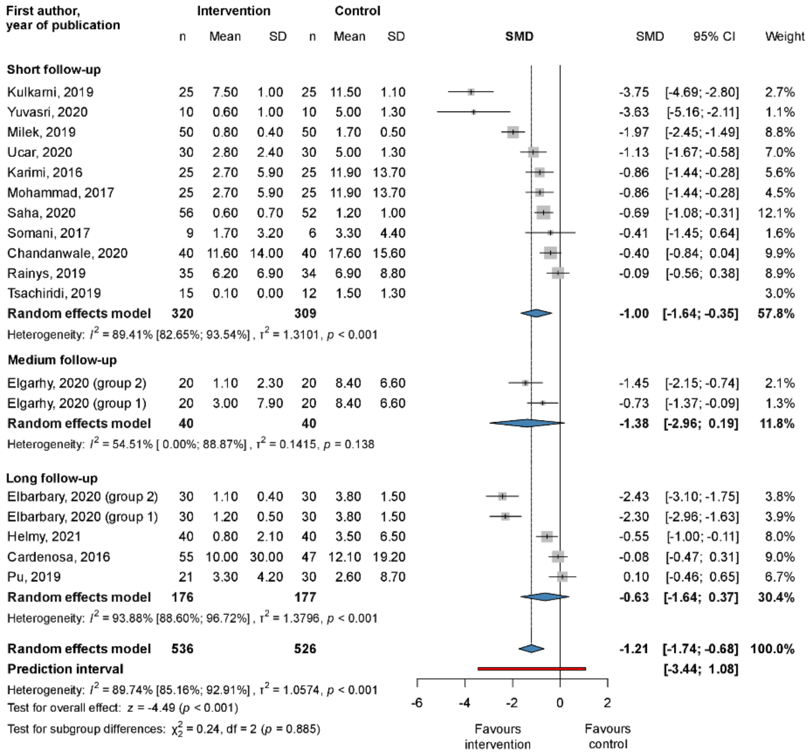

SD-standard deviation; SMD-standardized mean difference; CI-confidence interval

Figure S6. Forest plot for wound area reduction, subgrouping based on follow-up time.

First author, year of publication	Weight	Randomization process	Deviations from intended interventions	Missing outcome data	Measurement of the outcome	Selection of the reported result	Overall
Abd El Mabood, 2018	1	+	+	+	+	+	+
Ahmed, 2016	1	?	+	+	+	?	!
Amato, 2020	1	?	?	+	+	+	!
Anitua, 2008	1	?	+	?	+	+	!
Burgos-Alonso, 2018	1	?	+	+	+	+	!
Cardenosa, 2017	1	+	+	+	+	+	+
Chandanwale, 2020	1	?	+	+	+	+	!
de Oliviera, 2017	1	+	+	+	+	?	!
Driver, 2006	1	?	+	+	+	+	!
Elbarbary, 2020	1	+	+	+	+	+	+
Elgarhy, 2020	1	+	+	+	+	+	+
Elsaid, 2020	1	+	+	+	+	+	+
Game, 2018	1	+	+	+	+	+	+
Glukhov, 2017	1	?	+	+	+	+	!
Goda, 2018 1	1	+	+	+	+	+	+
Goda, 2018 2	1	+	+	+	+	+	+
Gude, 2019	1	?	?	+	+	?	!
Helmy, 2021	1	?	+	+	+	+	+
Hongying, 2020	1	+	?	+	?	+	!
Kakagia, 2007	1	?	?	+	?	+	!
Karimi, 2016	1	+	+	+	+	+	+
Kulkarni, 2019	1	?	+	+	+	+	+
Li, 2015	1	?	?	+	+	+	!
Milek, 2019	1	?	+	+	+	+	!
Mohammad, 2017	1	?	?	+	?	+	!
Moneib, 2018	1	?	+	+	+	+	+
Obolensky, 2014	1	?	?	+	+	+	!
Obolensky, 2017	1	?	+	+	+	+	!
Pu, 2019	1	+	+	+	+	+	+
Qin, 2019	1	?	?	+	?	+	!
Rainys, 2019	1	+	+	+	+	?	!
Ramos-Torrecillas, 2019	1	?	+	+	+	?	!
Saad Setta, 2011	1	?	?	+	?	+	!
Saha, 2020	1	+	+	+	+	+	+
Semenic, 2018	1	?	+	+	+	+	+
Senet, 2003	1	?	+	+	+	+	!
Singh, 2018	1	?	?	+	+	?	!
Singh, 2021	1	+	+	+	+	+	+
Sokolov, 2017	1	?	+	+	+	+	!
Somani, 2017	1	?	?	+	+	?	!
Tsachiridi, 2019	1	?	+	+	+	+	!
Tsai, 2019	1	?	+	+	+	+	+
Ucar, 2020	1	?	?	+	+	+	!
Yang, 2017	1	?	+	+	+	+	+
Yuvasri, 2020	1	?	+	+	+	?	!

+ Low risk
? Some concerns
— High risk

Figure S7. Risk of bias assessment of the included studies assessing the change of wound size [1–6,8–45], using the revised tool for assessing risk of bias in randomized trials (Rob 2).

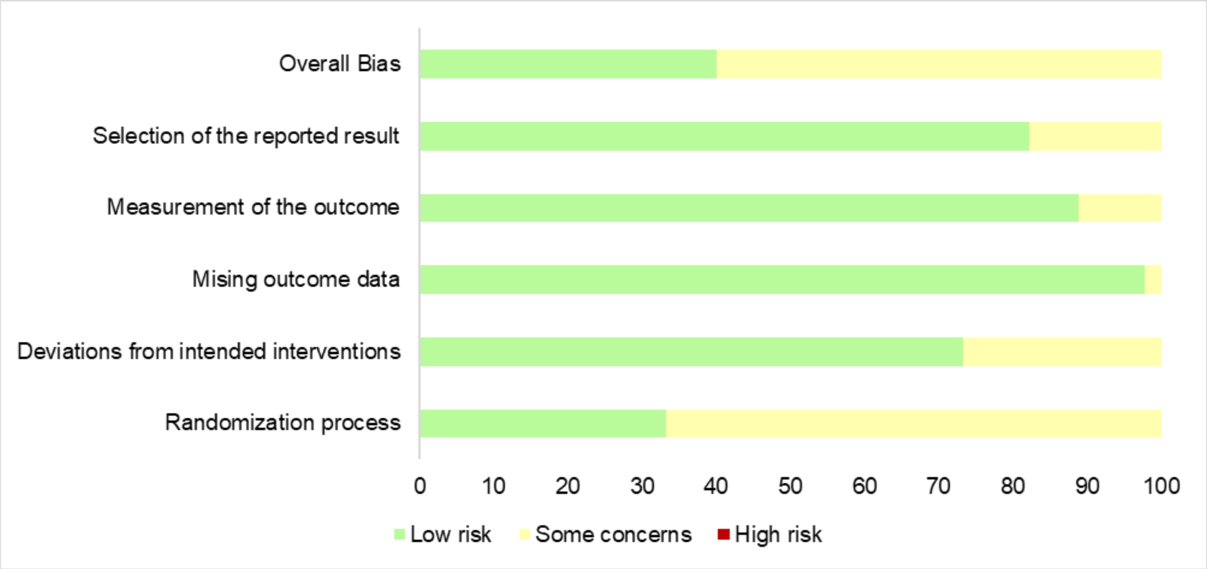

Figure S8. Risk of bias assessment of the included studies assessing the change of wound size [1–6,8–45], broken down to tools, shown in percentage.

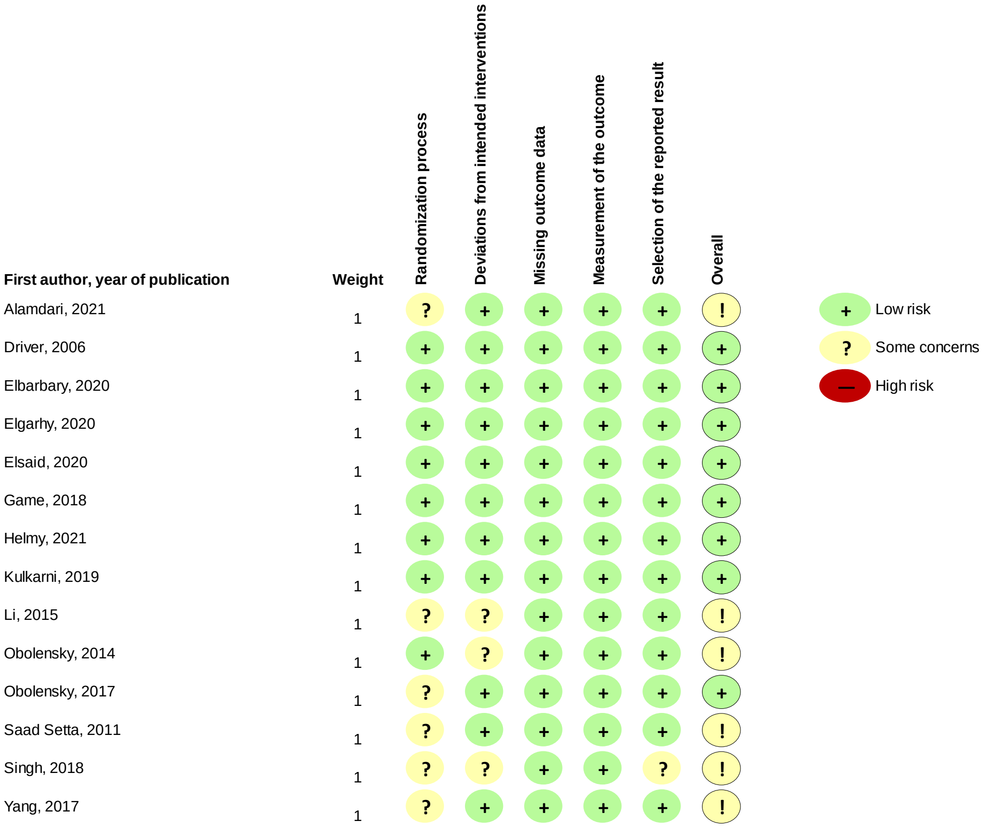

Figure S9. Risk of bias assessment of the included studies assessing healing time [9–13,18,22,23,27,28,33,38,44,46], using the revised tool for assessing risk of bias in randomized trials (Rob 2).

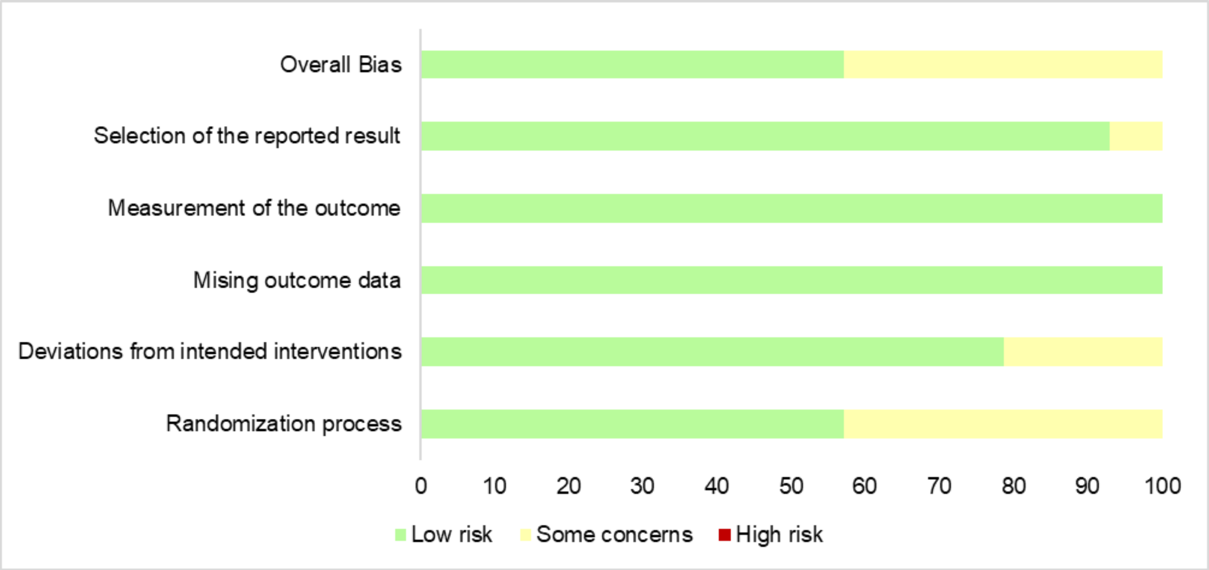

Figure S10. Risk of bias assessment of the included studies assessing healing time [9–13,18,22,23,27,28,33,38,44,46], broken down to tools, shown in percentage.

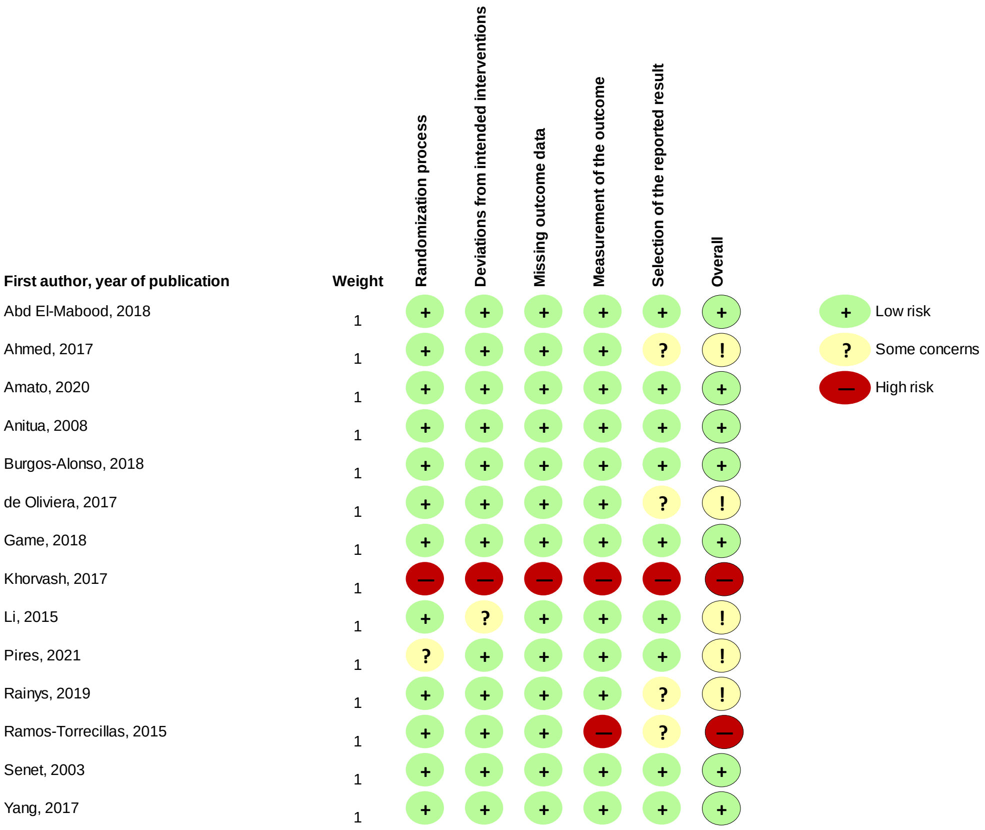

Figure S11. Risk of bias assessment of the included studies assessing infection rates [1–5,8,13,23,31,32,36,44,47,48], using the revised tool for assessing risk of bias in randomized trials (Rob 2).

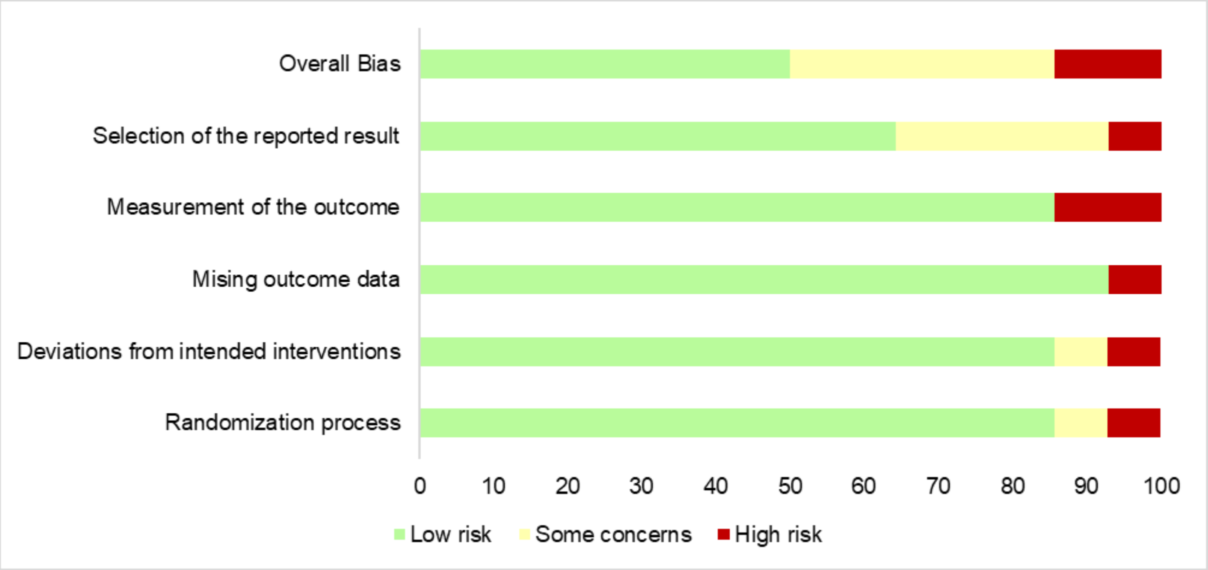

Figure S12. Risk of bias assessment of the included studies assessing infection rates [1–5,8,13,23,31,32,36,44,47,48], broken down to tools, shown in percentage.

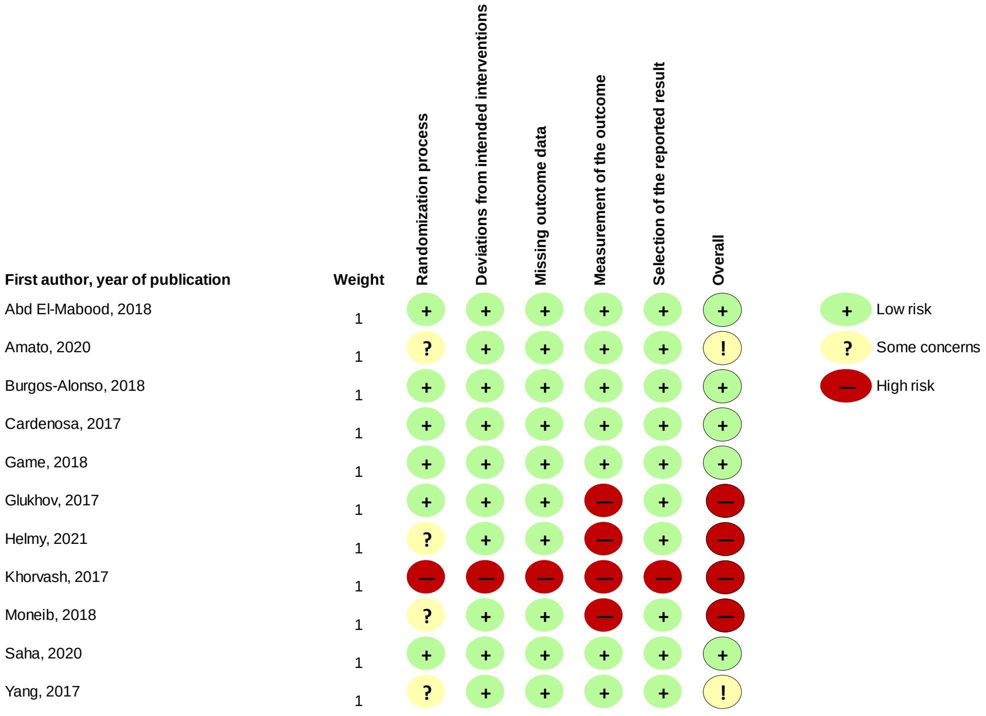

Figure S13. Risk of bias assessment of the included studies assessing pain [1,3,5,6,13,14,18,26,34,44,47], using the revised tool for assessing risk of bias in randomized trials (Rob 2).

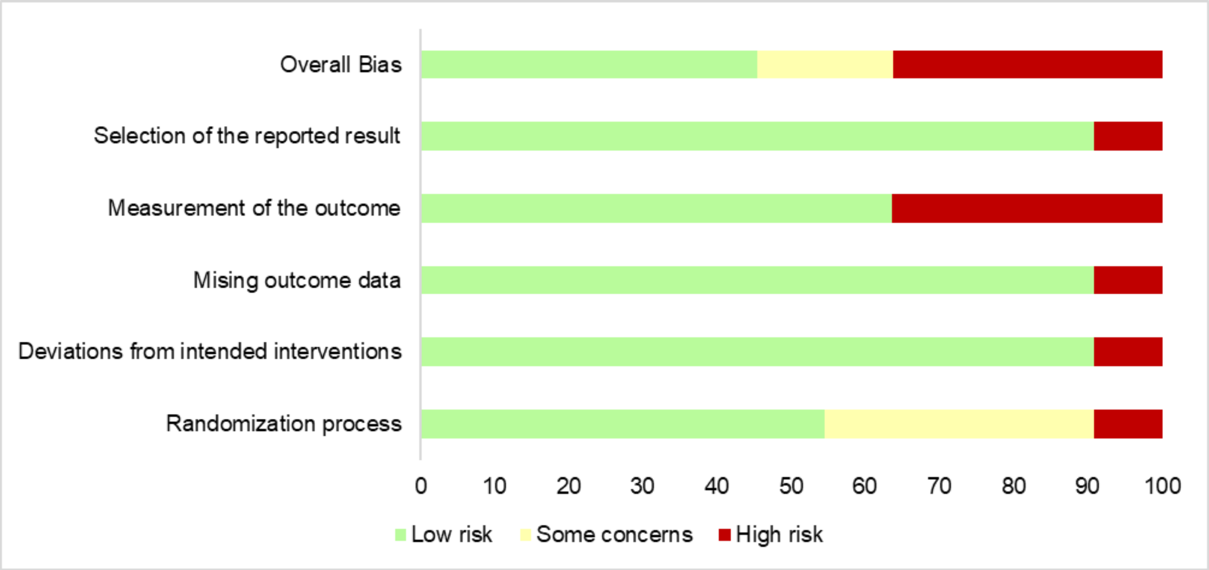

Figure S14. Risk of bias assessment of the included studies assessing pain [1,3,5,6,13,14,18,26,34,44,47], broken down to tools, shown in percentage.

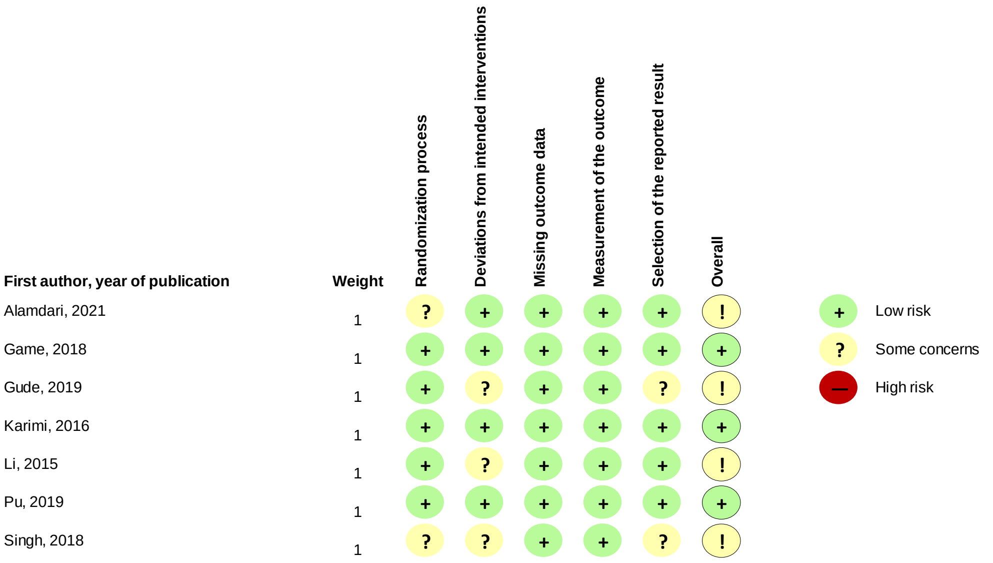

Figure S15. Risk of bias assessment of the included studies assessing amputation rates [13,17,21,23,29,37,46], using the revised tool for assessing risk of bias in randomized trials (Rob 2).

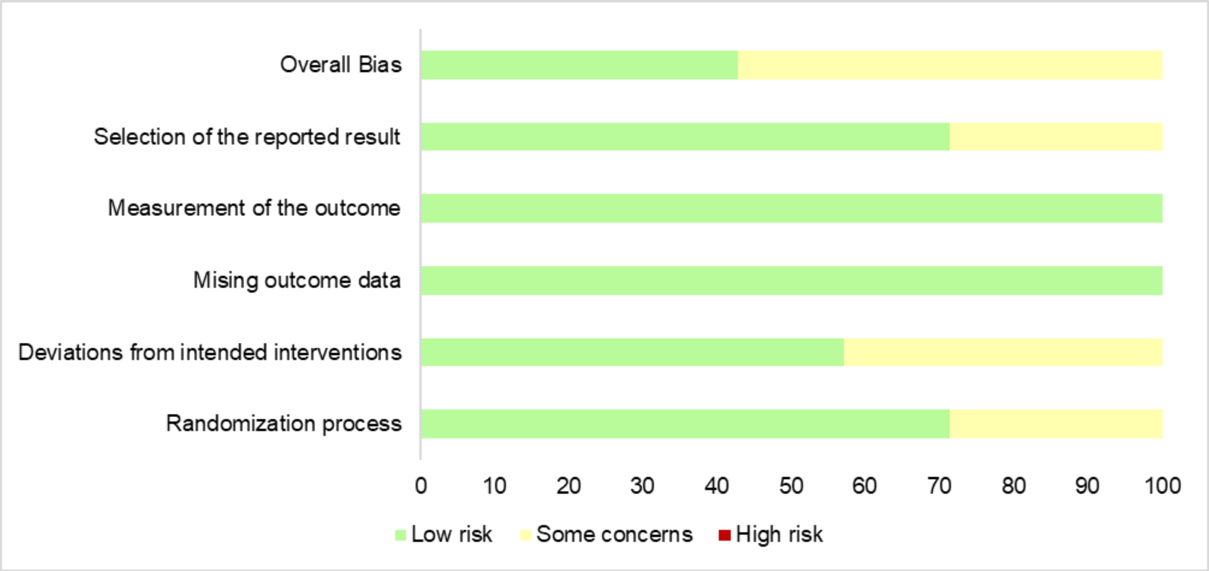

Figure S16. Risk of bias assessment of the included studies assessing amputation rates [13,17,21,23,29,37,46], broken down to tools, shown in percentage.

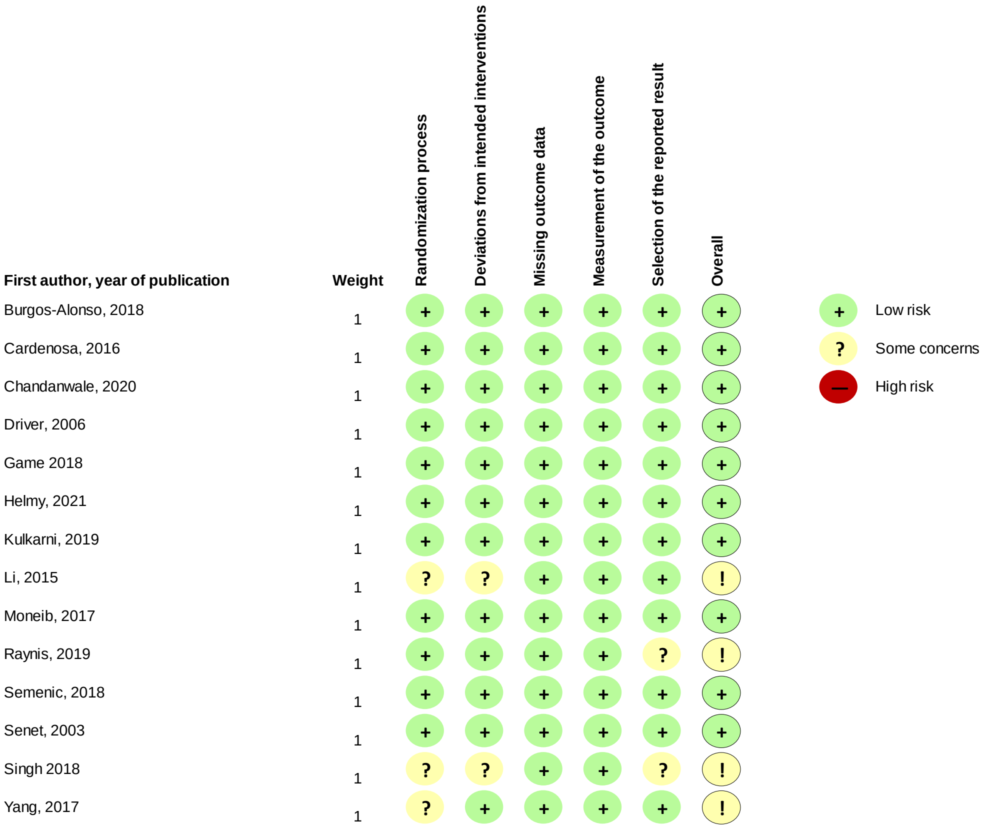

Figure S17. Risk of bias assessment of the included studies assessing adverse events [5–7,9,13,18,22,23,26,31,35,36,38,44], using the revised tool for assessing risk of bias in randomized trials (Rob 2).

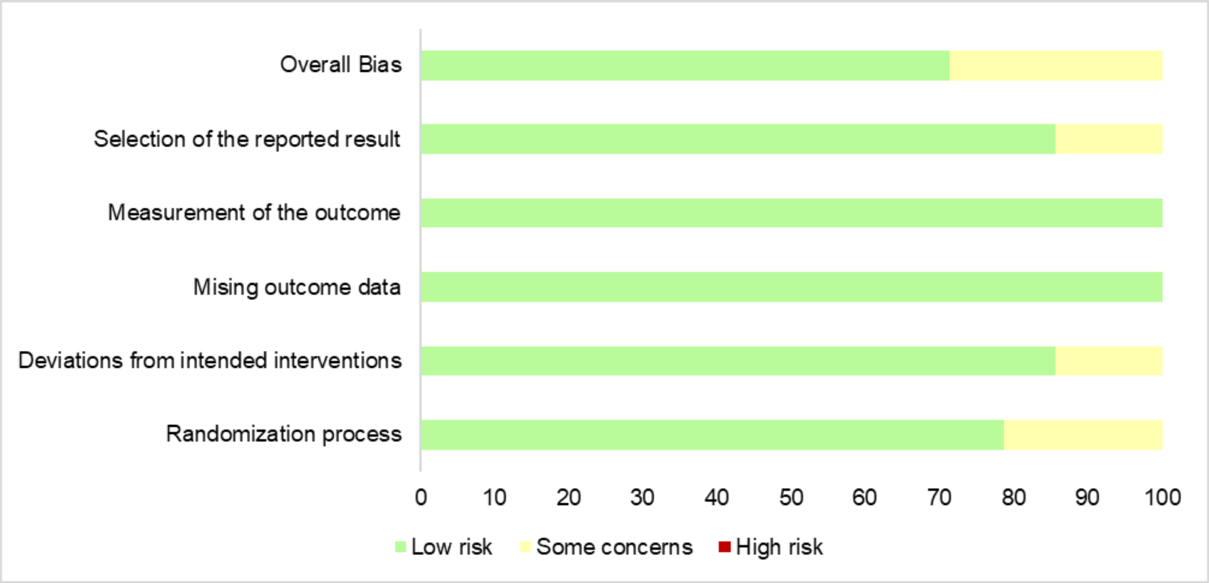

Figure S18. Risk of bias assessment of the included studies assessing adverse events [5–7,9,13,18,22,23,26,31,35,36,38,44], broken down to tools, shown in percentage.

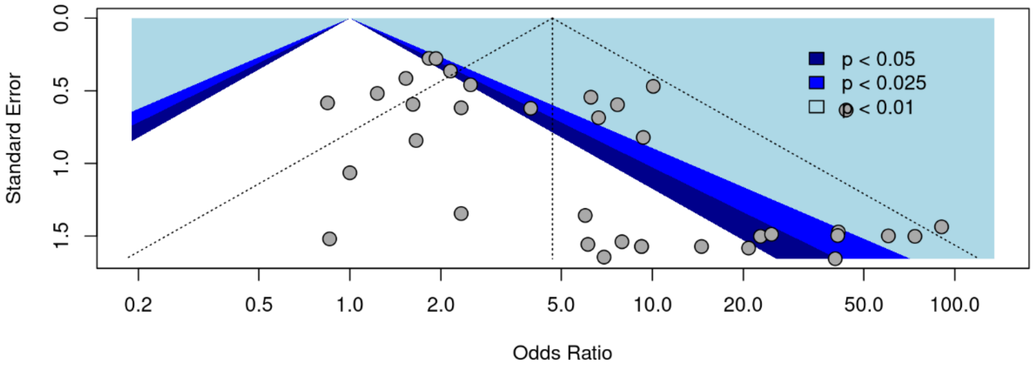

Figure S19. Funnel plot for complete closure.

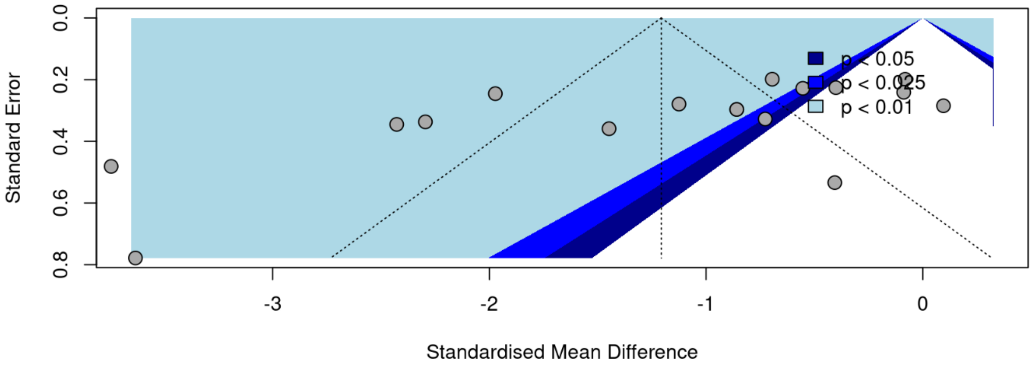

Figure S20. Funnel plot for the reduction of wound area.

Supplementary References

1. Abd El-Mabood, E. A. and H. E. Ali. Platelet-rich plasma versus conventional dressing: Does this really affect diabetic foot wound-healing outcomes? *Egyptian Journal of Surgery* **2018** *37*: 16-26.
2. Ahmed, M., S. A. Reffat, A. Hassan and F. Eskander. Platelet-rich plasma for the treatment of clean diabetic foot ulcers. *Annals of Vascular Surgery* **2017** *38*: 206-11.
3. Amato, B., M. A. Farina, S. Campisi, M. Ciliberti, V. Di Donna, A. Florio, A. Grasso, R. Miranda, F. Pompeo, E. Farina, *et al.* Cgf treatment of leg ulcers: A randomized controlled trial. *Open Medicine (Poland)* **2020** *14*: 959-67.
4. Anitua, E., J. J. Aguirre, J. Algorta, E. Ayerdi, A. I. Cabezas, G. Orive and I. Andia. Effectiveness of autologous preparation rich in growth factors for the treatment of chronic cutaneous ulcers. *Journal of Biomedical Materials Research - Part B Applied Biomaterials* **2008** *84*: 415-21.
5. Burgos-Alonso, N., I. Lobato, I. Hernandez, K. San Sebastian, B. Rodriguez, A. G. March, A. Perez-Salvador, V. Arce, A. Garcia-Alvarez, M. C. Gomez-Fernandez, *et al.* Autologous platelet-rich plasma in the treatment of venous leg ulcers in primary care: A randomised controlled, pilot study. *Journal of Wound Care* **2018** *27*: S20-S24.
6. Cardenosa, M. E., G. Dominguez-Maldonado and A. Cordoba-Fernandez. Efficacy and safety of the use of platelet-rich plasma to manage venous ulcers. *Journal of Tissue Viability* **2017** *26*: 138-43.
7. Chandanwale, K. A., C. C. Mahakalkar, A. K. Kothule and D. V. Khithani. Management of wounds of peripheral arterial disease using platelet rich plasma. *Journal of Evolution of Medical and Dental Sciences-Jemds* **2020** *9*: 2239-45.
8. de Oliveira, M. G., L. P. F. Abbade, H. A. Miot, R. R. Ferreira and E. Deffune. Pilot study of homologous platelet gel in venous ulcers. *Anais Brasileiros De Dermatologia* **2017** *92*: 499-504.
9. Driver, V. R., J. Hanft, C. P. Fylling and J. M. Beriou. A prospective, randomized, controlled trial of autologous platelet-rich plasma gel for the treatment of diabetic foot ulcers. *Ostomy/wound management* **2006** *52*: 68-70, 72, 74 passim.
10. Elbarbary, A. H., H. A. Hassan and E. A. Elbendak. Autologous platelet-rich plasma injection enhances healing of chronic venous leg ulcer: A prospective randomised study. *International Wound Journal* **2020** *17*: 992-1001.
11. Elgarhy, L. H., A. A. El-Ashmawy, A. E. Bedeer and A. M. Al-bahnasy. Evaluation of safety and efficacy of autologous topical platelet gel vs platelet rich plasma injection in the treatment of venous leg ulcers: A randomized case control study. *Dermatologic Therapy* **2020** *33*:
12. Elsaid, A., M. El-Said, S. Emile, M. Youssef, W. Khafagy and A. Elshobaky. Randomized controlled trial on autologous platelet-rich plasma versus saline dressing in treatment of non-healing diabetic foot ulcers. *World Journal of Surgery* **2020** *44*: 1294-301.
13. Game, F., W. Jeffcoate, L. Tarnow, J. L. Jacobsen, D. Whitham, E. F. Harrison, S. J. Ellender, D. Fitzsimmons, M. Londahl and I. I. T. T. LeucoPatch. Leucopatch system for the management of hard-to-heal diabetic foot ulcers in the uk, denmark, and sweden: An observer-masked, randomised controlled trial. *Lancet Diabetes & Endocrinology* **2018** *6*: 870-78.

14. Glukhov, A. A. and M. V. Aralova. The study of the effectiveness of the drug combination of collagen and platelet-rich plasma for the regional treatment of venous ulcers. *Research Journal of Pharmaceutical, Biological and Chemical Sciences* **2017** *8*: 2258-63.
15. Goda, A. A., M. Metwally, A. Ewada and H. Ewees. Platelet-rich plasma for the treatment of diabetic foot ulcer: A randomized, double-blind study. *Egyptian Journal of Surgery* **2018** *37*: 178-84.
16. Goda, A. A. Autogenous leucocyte-rich and platelet-rich fibrin for the treatment of leg venous ulcer: A randomized control study. *Egyptian Journal of Surgery* **2018** *37*: 316-21.
17. Gude, W., D. Hagan, F. Abood and P. Clausen. Aurix gel is an effective intervention for chronic diabetic foot ulcers: A pragmatic randomized controlled trial. *Advances in Skin & Wound Care* **2019** *32*: 416-26.
18. Helmy, Y., N. Farouk, A. Ali Dahy, A. Abu-Elsoud, R. Fouad khattab, S. Elshahat Mohammed, L. Abdullbary Gad, A. Altramsy, E. Hussein and A. Farahat. Objective assessment of platelet-rich plasma (prp) potentiality in the treatment of chronic leg ulcer: Rct on 80 patients with venous ulcer. *Journal of Cosmetic Dermatology* **2021** *20*: 3257-63.
19. Hongying, J., Z. Liang, Y. Xi, H. Jing, X. Xiaona, L. Zhengyan and H. Hongchen. Effect of platelet-rich plasma on pressure ulcers after spinal cord injury. *Chinese Journal of Tissue Engineering Research* **2020** *25*: 1149-53.
20. Kakagia, D. D., K. J. Kazakos, K. C. Xarchas, M. Karanikas, G. S. Georgiadis, G. Tripsiannis and C. Manolas. Synergistic action of protease-modulating matrix and autologous growth factors in healing of diabetic foot ulcers. A prospective randomized trial. *Journal of Diabetes and Its Complications* **2007** *21*: 387-91.
21. Karimi, R., M. Afshar, M. Salimian, A. Sharif and M. Hidariyan. The effect of platelet rich plasma dressing on healing diabetic foot ulcers. *Nursing and Midwifery Studies* **2016** *5*:
22. Kulkarni, S. R. and A. Chawla. Study of efficacy of platelet rich plasma dressing in management of chronic non-healing leg ulcers. *Journal of Evolution of Medical and Dental Sciences-Jemds* **2019** *8*: 1307-10.
23. Li, L., D. Chen, C. Wang, N. Yuan, Y. Wang, L. He, Y. Yang, L. Chen, G. Liu, X. Li, *et al.* Autologous platelet-rich gel for treatment of diabetic chronic refractory cutaneous ulcers: A prospective, randomized clinical trial. *Wound Repair and Regeneration* **2015** *23*: 495-505.
24. Milek, T., L. Nagraba, T. Mitek, W. Wozniak, K. Mlosek, W. Olszewski, P. Ciostek, J. Deszczynski, E. Kuchar and A. Stolarczyk. Autologous platelet-rich plasma reduces healing time of chronic venous leg ulcers: A prospective observational study. In *Advances in biomedicine*, M. Pokorski; Country, 2019; 1176, 109-17.
25. Mohammad, A., K. Rohangiz, S. Morteza, S. Alireza and A. Abolfazl. Comparison of platelet rich plasma and normal saline dressing effectiveness in the improvement of diabetic foot ulcers. *Journal of diabetic nursing* **2017** *5*: 246-55.
26. Moneib, H. A., S. S. Youssef, D. G. Aly, M. A. Rizk and Y. I. Abdelhakeem. Autologous platelet-rich plasma versus conventional therapy for the treatment of chronic venous leg ulcers: A comparative study. *Journal of Cosmetic Dermatology* **2018** *17*: 495-501.
27. Obolenskiy, V. N., D. A. Ermolova, L. A. Laberko and T. V. Semenova. Efficacy of platelet-rich plasma for the treatment of chronic wounds. *EWMA journal* **2014** *14*: 37-41.

28. Obolenskiy, V. N., D. A. Ermolova and L. A. Laberko. Clinical and economic effectiveness of the use of platelet-rich plasma in the treatment of chronic wounds. *Wound Medicine* **2017** *19*: 27-32.
29. Pu, D., X. Lei, W. Leng, Y. Zheng, L. Chen, Z. Liang, B. Chen and Q. Wu. Lower limb arterial intervention or autologous platelet-rich gel treatment of diabetic lower extremity arterial disease patients with foot ulcers. *Annals of Translational Medicine* **2019** *7*:
30. Qin, X. and J. Wang. Clinical study of local injection of autologous platelet-rich plasma in treatment of diabetic foot ulcer. *Zhongguo xiu fu chong jian wai ke za zhi = Zhongguo xiufu chongjian waike zazhi = Chinese journal of reparative and reconstructive surgery* **2019** *33*: 1547-51.
31. Rainys, D., A. Cepas, K. Dambrauskaite, I. Nedzelskiene and R. Rimdeika. Effectiveness of autologous platelet-rich plasma gel in the treatment of hard-to-heal leg ulcers: A randomised control trial. *Journal of Wound Care* **2019** *28*: 658-67.
32. Ramos-Torrecillas, J., O. García-Martínez, E. De Luna-Bertos, F. M. Ocaña-Peinado and C. Ruiz. Effectiveness of platelet-rich plasma and hyaluronic acid for the treatment and care of pressure ulcers. *Biological Research for Nursing* **2015** *17*: 152-58.
33. Saad Setta, H., A. Elshahat, K. Elsherbiny, K. Massoud and I. Safe. Platelet-rich plasma versus platelet-poor plasma in the management of chronic diabetic foot ulcers: A comparative study. *International Wound Journal* **2011** *8*: 307-12.
34. Saha, S., A. C. Patra, S. P. Gowda, N. Mondal, S. Rahaman, S. K. S. Ahmed, S. Debbarma, K. P. K. Vitthal, S. Sarkar, A. Sil, *et al.* Effectiveness and safety of autologous platelet-rich plasma therapy with total contact casting versus total contact casting alone in treatment of trophic ulcer in leprosy: An observer-blind, randomized controlled trial. *Indian J Dermatol Venereol Leprol* **2020** *86*: 262-71.
35. Semenič, D., T. Cirman, P. Rožman and D. M. Smrke. Regeneration of chronic wounds with allogeneic platelet gel versus hydrogel treatment: A prospective study. *Acta Clinica Croatica* **2018** *57*: 434-42.
36. Senet, P., F.-X. Bon, M. Benbunan, A. Bussel, R. Traineau, F. Calvo, L. Dubertret and C. Dosquet. Randomized trial and local biological effect of autologous platelets used as adjuvant therapy for chronic venous leg ulcers. *Journal of Vascular Surgery* **2003** *38*: 1342-48.
37. Singh, S. P., V. Kumar, A. Pandey, P. Pandey, V. Gupta and R. Verma. Role of platelet-rich plasma in healing diabetic foot ulcers: A prospective study. *Journal of Wound Care* **2018** *27*: 550-56.
38. Singh, G., D. Borah, G. Khanna and S. Jain. Efficacy of local autologous platelet-rich plasma in the treatment of pressure ulcer in spinal cord injury patients. *Cureus* **2021** *13*:
39. Sokolov, T., A. Manukova, S. Karakoleva, B. Valentinov and N. Petrova. Analysis of the results of applying the method platelet-rich plasma (prp) for the treatment of problematic skin wounds. *Journal of Imab* **2017** *23*: 1460-65.
40. Somani, A. and R. Rai. Comparison of efficacy of autologous platelet-rich fibrin versus saline dressing in chronic venous leg ulcers: A randomised controlled trial. *Journal of Cutaneous and Aesthetic Surgery* **2017** *10*: 8-12.
41. Tsachiridi, M., G. Galyfos, A. Andreou, A. Sianou, F. Sigala, G. Zografos and K. Filis. Autologous platelet-rich plasma for nonhealing ulcers: A comparative study. *Vascular Specialist International* **2019** *35*: 22-27.

42. Tsai, H. C., C. W. Lehman and C. M. Chen. Use of platelet-rich plasma and platelet-derived patches to treat chronic wounds. *Journal of Wound Care* **2019** *28*: 15-21.
43. Ucar, O. and S. Celik. Comparison of platelet-rich plasma gel in the care of the pressure ulcers with the dressing with serum physiology in terms of healing process and dressing costs. *International Wound Journal* **2020** *17*: 831-41.
44. Yang, L., L. Gao, Y. Lv and J. Wang. Autologous platelet-rich gel for lower-extremity ischemic ulcers in patients with type 2 diabetes. *International Journal of Clinical and Experimental Medicine* **2017** *10*: 13796-801.
45. Yuvasri, G. and R. Rai. Comparison of efficacy of autologous platelet-rich fibrin versus unna's paste dressing in chronic venous leg ulcers: A comparative study. *Indian Dermatology Online Journal* **2020** *11*: 58-61.
46. Alamdari, N. M., A. Sha, A. Mirmohseni and S. Besharat. Evaluation of the efficacy of platelet-rich plasma on healing of clean diabetic foot ulcers: A randomized clinical trial in tehran, iran. *Diabetes & Metabolic Syndrome-Clinical Research & Reviews* **2021** *15*: 621-26.
47. Khorvash, F., M. Pourahmad, N. Khoshchingol, M. Avijgan, M. Mohammadi and K. Sahebnazar. Comparing the effects of the platelet-rich plasma gel with wound therapeutic methods on the treatment of diabetic foot. *Journal of Isfahan Medical School* **2017** *35*: 1389-95.
48. Pires, B., B. G. R. Baptista de Oliveira, L. C. Bokehi, R. R. Luiz, B. T. F. Carvalho, R. F. Santana, P. Alfradique de Souza, G. Renato de Paula and L. A. Teixeira. Clinical and microbiological outcomes associated with use of platelet-rich plasma in chronic venous leg uclers: A randomized controlled trial. *Journal of wound, ostomy, and continence nursing : official publication of The Wound, Ostomy and Continence Nurses Society* **2021** *48*: 292-99.
